# Leukocyte Expression of Type 1 and Type 2 Purinergic Receptors and Pro-Inflammatory Cytokines during Total Sleep Deprivation and/or Sleep Extension in Healthy Subjects

**DOI:** 10.3389/fnins.2017.00240

**Published:** 2017-05-02

**Authors:** Mounir Chennaoui, Pierrick J. Arnal, Catherine Drogou, Damien Leger, Fabien Sauvet, Danielle Gomez-Merino

**Affiliations:** ^1^Fatigue and Vigilance team, Neuroscience and Operational Constraints Department, French Armed Forces Biomedical Research Institute (IRBA)Brétigny-sur-Orge, France; ^2^VIFASOM team (EA 7330), Paris Desacrtes University, Sorbonne Paris CitéHôtel Dieu, Paris, France; ^3^Centre du Sommeil et de la Vigilance, Hôtel Dieu, Assistance publique - Hôpitaux de ParisParis, France

**Keywords:** sleep deprivation, adenosine and purinergic receptors, gene expression, leukocytes

## Abstract

The purinergic type P1 (adenosine A_1_ and A_2A_) receptors and the type P2 (X7) receptor have been suggested to mediate physiological effects of adenosine and adenosine triphosphate on sleep. We aimed to determine gene expression of A_1_R (receptor), A_2A_R, and P2RX_7_ in leukocytes of healthy subjects during total sleep deprivation followed by sleep recovery. Expression of the pro-inflammatory cytokines IL-1β and TNF-α were also determined as they have been characterized as sleep regulatory substances, via P2RX_7_ activation. Blood sampling was performed on 14 young men (aged 31.9 ± 3.9) at baseline (B), after 24 h of sleep deprivation (24 h-*SD*), and after one night of sleep recovery (R). We compared gene expression levels after six nights of habitual (22.30–07.00) or extended (21.00–07.00) bedtimes. Using quantitative real-time PCR, the amount of mRNA for A_1_R, A_2A_R, P2RX_7_, TNF-α, and IL-1β was analyzed. After 24 h-*SD* compared to B, whatever prior sleep condition, a significant increase of A_2A_R expression was observed that returned to basal level after sleep recovery [day main effect, *F*_(2, 26)_ = 10.8, *p* < 0.001]. In both sleep condition, a day main effect on P2RX_7_ mRNA was observed [*F*_(2, 26)_ = 6.7, *p* = 0.005] with significant increases after R compared with 24 h-*SD*. TNF-α and IL-1β expressions were not significantly altered. Before 24 h-*SD* (baseline), the A_2A_R expression was negatively correlated with the latency of stage 3 sleep during the previous night, while that of the A_1_R positively. This was not observed after sleep recovery following 24 h-*SD*. This is the first study showing increased A_2A_R and not A_1_ gene expression after 24 h-*SD* in leukocytes of healthy subjects, and this even if bedtime was initially increased by 1.5 h per night for six nights. In conclusion, prolonged wakefulness induced an up-regulation of the A2A receptor gene expression in leukocytes from healthy subjects. Significant correlations between baseline expression of A_1_ and A_2A_ receptors in peripheral cells and stage 3 sleep suggested their involvement in mediating the effects of adenosine on sleep.

## Introduction

Adenosine is a metabolic intermediate of the energy-rich molecule adenosine-tri-phosphate (ATP) and a pleiotropic bioactive molecule with potent neuromodulatory properties because of its ability to cross the blood-brain barrier; it was proposed as a signaling molecule between the periphery and the brain (Chiu and Freund, 2014). Adenosine receptors are ubiquitously distributed throughout brain, heart, immune, and inflammatory cells and many other tissues. In the central nervous system (CNS), adenosine plays important functions such as modulation of neurotransmitter release, synaptic plasticity, and neuroprotection in ischemic, hypoxic, and oxidative stress events (Rodrigues et al., 2015). Numerous studies indicate that endogenous adenosine is a candidate for the sleep regulation and it induced sleep after prolonged wakefulness (Basheer et al., 2004). Moreover, adenosine interplays with several neurotransmitters that regulate sleep (Weber and Dan, 2016). Adenosine promotes sleep, at least in part, by inhibiting wakefulness-promoting neurons localized to the basal forebrain, lateral hypothalamus, and tuberomammillary nucleus of the posterior hypothalamus. There are two forms of sleep, non-rapid eye movement (NREM) sleep and rapid eye movement (REM) sleep. In humans, NREMS occupies about 80% and REMS about 20% of sleep time. These two stages alternate with each other with about a 90–100 min periodicity, with more NREM sleep occurring during the first half and more REM sleep occurring during the second half of the night (Krueger et al., 2008). The formation of adenosine in the brain changes in activity-dependent manner and is linked to the intracellular depletion of ATP (Krueger et al., 2013). The sleep inducing effects of extracellular adenosine are mediated through only two of the four adenosine receptor subtypes (A_1_, A_2A_, A_2B_, and A_3_): the inhibitory G protein-coupled adenosine A1 receptor (A_1_R) and the excitatory G protein-coupled adenosine A2 receptor (A_2_R) (Landolt et al., 2012). This author has suggested that both A_1_and A_2A_ receptor subtypes probably mediate the effects of adenosine on vigilance and sleep, and these effects appear to be brain regions and receptor-dependent. Adenosine seems to promote sleep by simultaneously activating sleep-active neurons through A_2A_R and suppressing wake-active neurons through A_1_R (Watson et al., 2010). The A_1_R activation in histaminergic neurons of the tuberomammillary nucleus increases NREM sleep (Oishi et al., 2008) while the activation of A_2A_R in the pontine reticular formation increases acetylcholine release and NREM sleep (Coleman et al., 2006). Extracellular adenosine levels in basal forebrain and frontal cortex increased during 6 or 11 h of sleep deprivation but not in four other subcortical structures, and then decreased during recovery sleep (Porkka-Heiskanen et al., 2000; Kalinchuk et al., 2011). After 5 days of sleep restriction, A_1_R mRNA levels and density were increased in the basal forebrain region and several brain regions respectively, whereas A_2A_R mRNA levels and density were reduced in the frontal cortex and olfactory tubercle (Kim et al., 2012, 2015). Total sleep deprivation also upregulates A_1_R mRNA levels in the rat basal forebrain and increased its density in cortical and subcortical brain regions (Basheer et al., 2007; Elmenhorst et al., 2009). In the human brain, a single night of total sleep deprivation leads to a significant increase of the A_1_R binding (Elmenhorst et al., 2007). The same research group recently showed that variation of A_1_R regional availability is associated with the A_1_R and A_2A_R gene polymorphisms in the human brain (Hohoff et al., 2014). In addition, a role for the A_2A_R gene polymorphism has been particularly related to be an important determinant of psychomotor vigilance in rested and in acute and chronic sleep-deprived state (Bodenmann et al., 2012; Rupp et al., 2013). Indeed several studies have previously shown that sleep deprivation or restriction is associated with increased daytime sleepiness, decreased cognitive and physical performance, increased inflammatory markers (pro-inflammatory cytokines IL-1β and TNF-α) and hormonal/metabolic disturbances (Rupp et al., 2009; Chennaoui et al., 2011, 2015; Arnal et al., 2015, 2016; Irwin et al., 2015; Sauvet et al., 2015).

During wakefulness, the increase of extracellular ATP in brain may either be hydrolyzed to adenosine or activate purine type 2 receptors such as P2X_7_ receptors (P2RX_7_) that affect sleep directly or indirectly through cytokines release or other sleep regulatory substances (Krueger, 2008; Krueger et al., 2010). The gene expression of P2RX_7_ has been evidenced in immune cells (North, 2002) and one study has shown increased expression in peripheral monocytes after 24 h of sleep deprivation in healthy young volunteers (Backlund et al., 2012).

The A_1_ and A_2A_ and P2X_7_ receptors are expressed in human leukocytes and were reported to play a central role in mechanisms of inflammation associated to various neurological pathologies (Fortin et al., 2006; Varani et al., 2010; Chiu and Freund, 2014; Casati et al., 2016; Krügel, 2016). Moreover, leukocytes have been proposed as a useful peripheral model to study inflammatory processes in the CNS (Sullivan et al., 2006; Varani et al., 2010; Light et al., 2012).

In this study, we determined for the first time whether advancing bedtime by 1.5 h per night over six nights (i.e., going to bed at 21.00 vs. 22.30 and getting up at 07.00 in both conditions) influences A_1_R, A_2A_R, and P2RX_7_ mRNA levels in leukocytes of healthy subjects during total sleep deprivation (TSD) followed by one night of sleep recovery. The gene expressions of the pro-inflammatory somnogenic cytokines IL-1β and TNF-α were also determined. We hypothesized that longer bedtime for sleep and/or sleep deprivation may altered peripheral purine type 1 and type 2 receptors.

## Methods

### Subjects

Fourteen healthy men, aged 31.4 ± 3.9 years (mean ± *SD*), with a body mass index (BMI) of 24.0 ± 2.0 kg/m^2^ were included in the study after providing their written informed consent. The ethics committee of the Hotel Dieu Hospital—Ile de France 1 (Paris) and the French National Agency for the Safety of Medicines and Health Products (ANSM) approved the protocol (N°ID RCB: 2013-A01403-42), which was conducted according to the principles expressed in the Declaration of Helsinki of 1975 as revised in 2001. All subjects underwent a detailed medical history and examination including an electrocardiogram at rest, performed by a physician. Subjects were excluded if they were shift-workers, smokers, consumers of more than three alcoholic beverages weekly and of more than 400 mg of caffeine per day (i.e., about eight caffeinated sodas or 3–4 cups of coffee), with a BMI > 28 kg/m2, and taking medication. Exclusion criteria also included: subjects with excessive daytime somnolence (Epworth Sleepiness Scales > 11; Johns, 1961), sleep complaints (Pittsburgh Sleep Quality Index > 5; Buysse et al., 1989), who could not be considered as an intermediate chronotype or moderately morning type on the Horne and Ostberg questionnaire (<42 and >69; Horne and Ostberg, 1976), or who scored ≥13 on the Beck Depression Inventory (Beck et al., 1961), or ≥13 on the Hospital Anxiety and Depression Scale (HADS; Zigmond and Snaith, 1983).

### Protocol

The TSD experimental protocol took place at the Hôtel Dieu Hospital (Paris, France) where subjects were housed individually in a temperature-controlled bedroom (24 ± 1°C). The total sleep deprivation started at 07:00 after one night of sleep in the laboratory and finished the day after at 21:00 (i.e., 38 h of continuous awakening). After one night of sleep recovery, subjects left the laboratory in the afternoon. During the TSD protocol, the sleep of subjects was continuously monitored by an ultra-miniaturized polysomnography PSG in order to limit patient discomfort (Actiwave, CamNtech LtD, Cambridge, UK). Laboratory illumination was maintained at 150–200 lux during the entire period of sleep loss. When not engaged in any specific testing or meal, subjects were allowed to read, watch videos, or play games, or converse with the staff or visitors. Subjects were prohibited from exercise, caffeine, tobacco, alcohol, and other psychoactive substances 24 h before and during the study. Meals and caloric intake were standardized for all subjects (2700 kcal/day). Water was allowed *ad libitum*.

Blood samples were collected at 07:00 in fasting state, subjects lying down on bed. They were collected at baseline (B), after 24 h of sleep deprivation (24 h-*SD*), and after the recovery sleep (R). For mRNA quantification, blood was collected in PAXgene blood RNA tubes (PreAnalytix, GmbH, 8634 Hombrechtikon, CH), and tubes were stored at −80°C.

**Figure 1:**
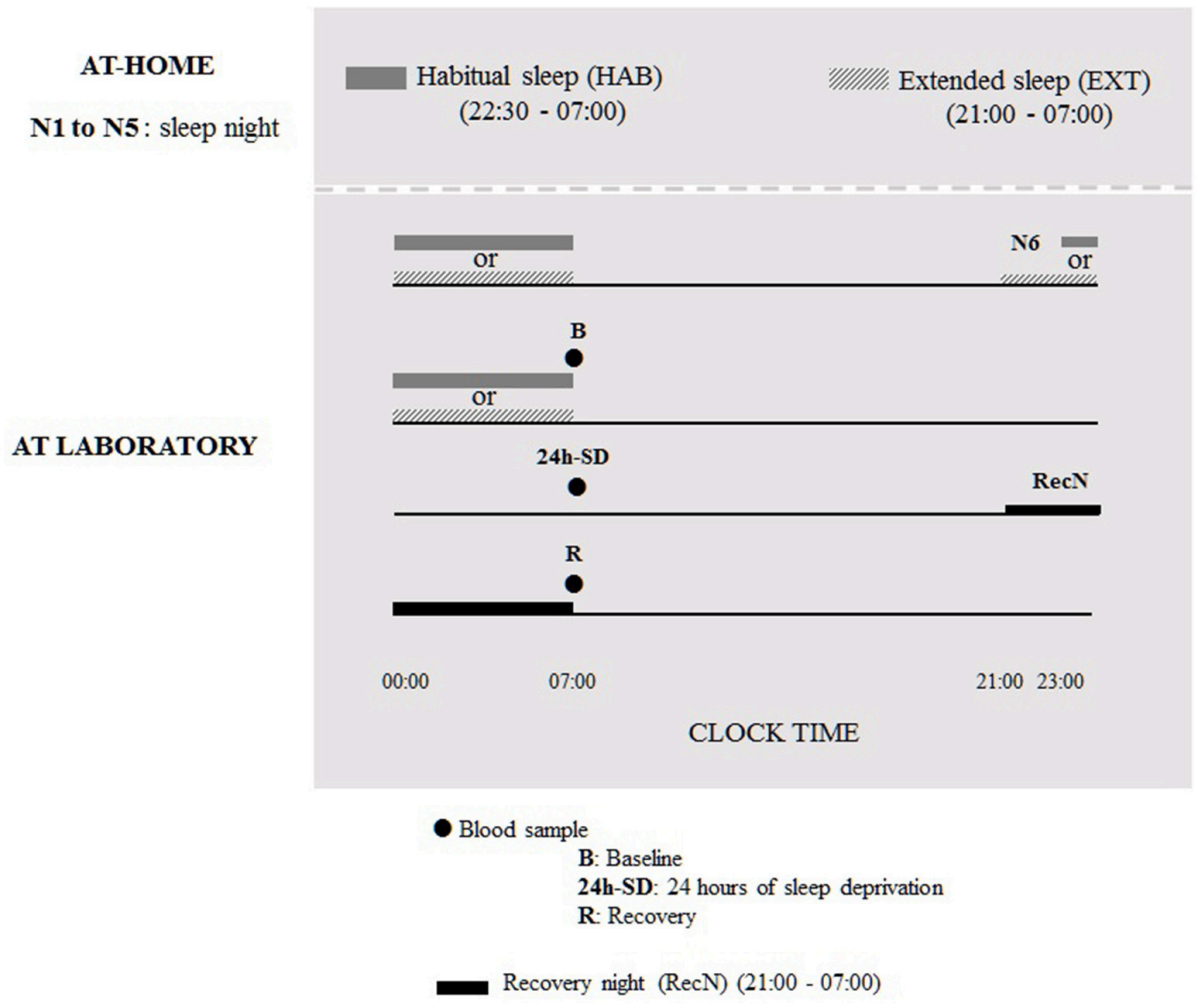
**Design of experimental protocol**.

During six nights before the TSD protocol, subjects were under two counterbalanced sleep conditions (cross-over design): habitual sleep condition (HAB) (22.30–07.00 h) or extended sleep condition (EXT) (21.00–07.00 h). The sleep was recorded by an ultra-miniaturized PSG. The washout period between the two experimental conditions was 6 weeks. In the 21.00–07.00 h sleep condition, subjects were allowed to read a little if needed to sleep, although cell phone/tablet use was prohibited, and they were instructed to turn off lights for sleeping and not to take any naps during the day. In addition, the use of low and indirect lighting was recommended before going to bed. In both sleep conditions, volunteers maintained a wake-up time of 07:00 to accustom them to the waking time of 07:00 used during the TSD protocol in laboratory. Volunteers were allowed to maintain their usual lifestyles but needed to return the PSG equipment to the laboratory every morning. The first five nights were conducted at home and the sixth night was at the laboratory under HAB or EXT sleep condition; for the night of sleep recovery subjects went to bed at 21:00 and get up at 07:00 (Figure 1).

### Measurements

#### Night-time sleep assessment

An ultra-miniaturized PSG (Actiwave, CamNtech LtD, Cambridge, UK) provides continuous monitoring for six EEG (F3, C3, O1 and F4, C4, O2), two electrocardiograms and two electrooculograms (outer canthus of each eye), and two electromyograms (chin). Contralateral mastoid leads served as references for all unipolar measurements (electroencephalograms and electrooculograms). Bio-electrical EEG signals were digitized at a sampling frequency of 200 Hz with a 16 bit quantization between −500 and 500 μV, within a bandwidth of 0–48 Hz. PSG data were scored by two trained research technicians in accordance with the American academy of sleep medicine criteria (Iber et al., 2007) using Somnologica software (TM, Medcare®, Reykjavik, Iceland). Duration of the different sleep stages [sleep stages 1, 2, 3, and rapid eye movement (REM) sleep], latencies of stage 3 and REM sleep, wakefulness after sleep onset (WASO), sleep efficiency, and sleep latency were determined.

#### Quantification of blood cells

Leukocyte counts and subsets were determined on the automated hematology system XE 5000 (SYSMEX Mundelein, IL 60060, US).

#### Gene expression analysis

##### mRNA isolation and reverse transcription

RNA from blood collected in PAXgene RNA tubes was extracted according to the manufacturer's instructions. RNA quantity was measured with a Nanodrop spectrophotometer ND-1000 (Thermo Scientific). RNA was immediately stored at −80°C. Reverse transcription was performed using the RT2 HT First Strand kit (Qiagen). The reaction was carried out using 0.5 μg of RNA. The cDNA was stored at –80°C until use.

##### Real time PCR

PCR was carried out with 96-well plate's customed RT2 Profiler Arrays in combination with RT2 SYBR Green mastermix (SA Biosciences Qiagen, Venlo, Netherlands) using 1 μL of cDNA in a final volume of 25 μL. The cDNA sequences (human) for TNF-α, IL-1β, and P2RX_7_, A_1_R and A_2A_R were from GenBank (accession numbers NM_000594, NM_000576, NM_002562, NM_000674, and NM_000675, respectively), as were those of the three reference genes HMBS, H6PD, and HPRT1 (accession numbers NM_000190, NM_004285, and NM_000194). Reactions were performed on a LightCycler 480 (96-well-block) and the crossing point values were calculated using LightCycler Software v3.5 (Roche Applied Science, Mannheim, Germany). Amplification specificity was checked using melting curve analysis following the manufacturer's instructions. The genomic DNA control (GDC), the reverse-transcription control (RTC) and the positive PCR control (PPC) were tested for each sample for genomic DNA contamination, reverse transcription efficiency, and the polymerase chain reaction, respectively. The normalization was performed by geometric averaging of the three housekeeping genes (HMBS, H6PD, and HPRT1). The mean normalized mRNA (i.e., mRNA/mRNA_housekeeping genes_) ± SEM values are presented.

### Statistics

All data in text and figures are presented as mean ± standard error of the mean (SEM). Statistical analyses were performed using Statistica 6.0, StatSoft Inc., Maisons-Alfort, France.

Two-way repeated measures ANOVAs (condition × night) were conducted on sleep parameters. Two-way ANOVAs measures (sleep condition × day) were conducted on blood parameters. When the ANOVA revealed significant interactions or main effect, a Tukey *post-hoc* test was used to identify differences. Pearson's test was employed for the correlation analysis. Statistical significance was set at *p* < 0.05.

## Results

### Blood cells count and gene expression analysis

In the two sleep conditions (22.30–07.00 and 21.00–07.00) no significant difference (sleep condition and day main effects) in leukocyte counts was found (B, 24 h-*SD, R*: 6.9 ± 1.6, 7.2 ± 1.5, and 7.4 ± 1.5 for HAB and 7.3 ± 2.1, 7.6 ± 1.8, 7.2 ± 1.9 for EXT, expressed as cells × 10^9^). Lymphocyte, monocyte and neutrophil counts were not significantly changed either during sleep deprivation or between sleep conditions (data not shown).

**Figure 2:**
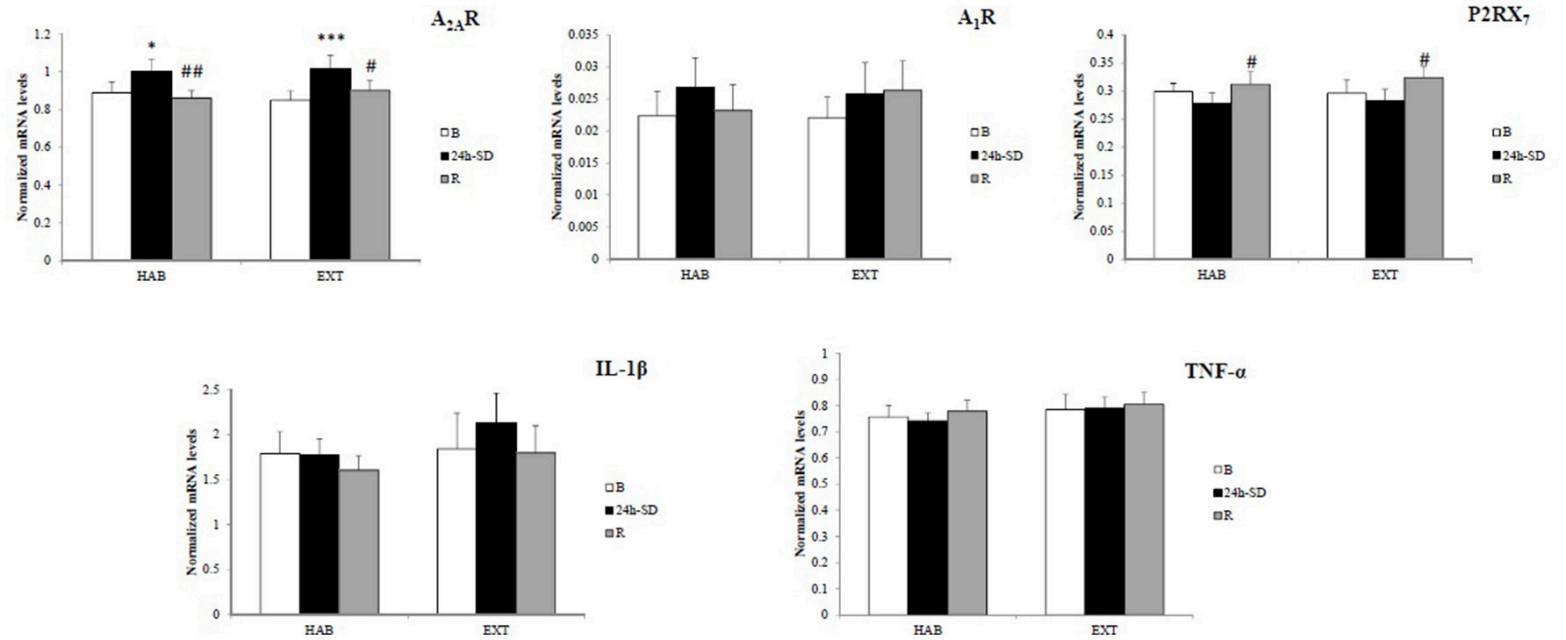
**Normalized mRNA levels of adenosine (A_**2A**_ and A_**1**_) and type 2 purinergic (P2X_**7**_) receptors, and IL-1β and TNF-α in leukocytes of healthy men in habitual (HAB) and extended (EXT) sleep conditions (respectively, 22.30–07.00 and 21.00–07.00 bedtimes), at baseline (B), after 24 h of sleep deprivation (24 h-***SD***) and after one night of sleep recovery (R)**. *N* = 14. ^*^Significantly different from baseline (B), ^*^p < 0.05, ^***^p < 0.001. #Significantly different from 24-*SD*, #*p* < 0.05 and ##*p* < 0.01.

Figure 2 shows amount of mRNA encoding A_2A_R, A_1_R, P2RX_7_, IL-1β, and TNF-α. In the two sleep conditions (HAB and EXT), a significant increase in amount of mRNA encoding A_2A_R was observed after 24 h-*SD* that returned to their basal concentrations after recovery sleep [day main effect, *F*_(2, 26)_ = 10.8, *p* < 0.001].

In the two sleep conditions (HAB and EXT), P2RX_7_ mRNA expression levels increased significantly after the night of sleep recovery compared to 24 h-*SD* [day main effect, *F*_(2, 26)_ = 6.7, *p* = 0.005].

No significant difference (sleep condition and day main effects) in gene expression of A_1_R [*F*_(1, 13)_ = 0.11, *p* = 0.74; *F*_(2, 26)_ = 1.69, *p* = 0.20], TNFα [*F*_(1, 13)_ = 1.70, *p* = 0.21; *F*_(2, 26)_ = 0.57, *p* = 0.57], and IL-1β [*F*_(1, 13)_ = 0.95, *p* = 0.35; *F*_(2, 26)_ = 2.53, *p* = 0.09] was found.

The correlation analysis showed that mRNA levels of A_2A_R and TNF-α were significantly and positively correlated at B, 24 h-*SD* and R after considering all subjects in the two sleep conditions (*r* = 0.564, *r* = 0.425, and *r* = 0.503, *p* < 0.05, respectively).

### Night-time sleep assessment

Table 1 summarizes the sleep parameters during the sixth night before TSD (N6) and the night of sleep recovery (RecN) under HAB or EXT sleep conditions. Total sleep time and sleep stages 1, 2, and REM were significantly longer in the EXT compared to HAB sleep condition, while no difference was observed for sleep stage 3, stage 3 and REM latencies, sleep efficiency, sleep latency, and WASO. There was no difference between sleep conditions for any of the PSG parameters during the RecN.

**Table 1:** **Sleep parameters during the night before total sleep deprivation (N6) and the night of sleep recovery (RecN) in habitual and extended sleep condition**.

		**N6**	**RecN**	**ANOVA**		
				**Sleep condition *F*_(1, 12)_**	**Night *F*_(1, 12)_**	**Interaction *F*_(1, 12)_**
Total sleep time (min)	HAB	411 ± 8	543 ± 6^***^	51^$$$^	173.7^$$$^	43.5^$$$^
	EXT	513 ± 10^###^	545 ± 3			
Stage 1 (min)	HAB	20 ± 2	7 ± 1^***^	14.3^$$^	47.4^$$$^	4.20
	EXT	34 ± 4^###^	9 ± 2^***^			
Stage 2 (min)	HAB	175 ± 9	193 ± 9	20.3^$$$^	0.20	6.8^$$^
	EXT	222 ± 11^###^	195 ± 11			
Stage 3 (min)	HAB	132 ± 7	231 ± 13^***^	0.60	188.4^$$$^	1.80
	EXT	149 ± 12	222 ± 10^***^			
Stage 3 latency	HAB	38 ± 5	12 ± 2^**^	3.40	57.8^$$$^	0.01
	EXT	45 ± 5	20 ± 4^**^			
REM (min)	HAB	84 ± 6	112 ± 9	9.6^$$^	14.2^$$^	1.10
	EXT	109 ± 7^#^	119 ± 4			
REM latency	HAB	129 ± 9	162 ± 22	0.22	0.71	2.41
	EXT	159 ± 14	144 ± 14			
Sleep efficiency (%)	HAB	85 ± 2	94 ± 1^***^	0.10	47.3^$$$^	0.10
	EXT	85 ± 2	95 ± 1^***^			
Sleep latency (min)	HAB	25 ± 4	6 ± 1^***^	0.10	70.7^$$$^	0.10
	EXT	25 ± 4	6 ± 2^***^			
WASO (min)	HAB	47 ± 6	27 ± 6^*^	1.10	17.1^$$$^	4.00
	EXT	63 ± 8	24 ± 4^*^			

ANOVA effect:

$$ *p < 0.01*,

$$$ *p < 0.001*.

N6 vs. RecN:

* *p < 0.05*,

** *p < 0.01*,

*** *p < 0.001*.

HAB vs. EXT:

# *p < 0.05*,

### *p < 0.001*.

*WASO, Wake after sleep onset; REM, Rapid eye movement*.

In the HAB sleep condition, total sleep time, sleep stage 1, stage 3 and stage 3 latency, sleep efficiency and latency, and WASO were higher after RecN compared to N6. In the EXT sleep condition, sleep stage 1, stage 3 and stage 3 latency, sleep efficiency and latency, and WASO were higher after RecN compared to N6.

We performed the correlation analysis between A_1_R, A_2A_R, and TNF-α mRNA levels at baseline (i.e., B) and the sleep characteristics of the N6 night. The analysis was also performed between mRNA levels after sleep recovery (i.e., R) and the sleep characteristics of the RecN. At baseline, the latency of stage 3 sleep correlated with A_1_ and A_2A_R gene expression in an opposite way. In the EXT condition, we find negative correlation with A_2A_R mRNA levels, and positive correlation with A_1_R mRNA levels in the HAB condition. At recovery, the only one significant negative correlation was between the TNF-α mRNA and the latency of stage 3 sleep in the EXT sleep condition (Table 2). When data of HAB and EXT sleep conditions were pooled (*N* = 52), Pearson rank correlation coefficients between A_2A_R and A_1_R expression and latency of stage 3 sleep were respectively: −0.406 and 0.435, *p* < 0.05.

**Table 2:** **Pearson rank correlation coefficients between mRNA levels of A_**1**_R, A_**2**_AR, and TNF-α at baseline and recovery and sleep parameters of the previous night (N6 or RecN)**.

**BASELINE**			
Stage 3 sleep latency	HAB	0.703^*^	A_1_R
N6	EXT	0.145	A_1_R
	HAB	−0.182	A_2A_R
	EXT	−0.650^*^	A_2A_R
	HAB	−0.029	TNF-α
	EXT	−0.720^*^	TNF-α
Sleep onset latency	HAB	0.612^*^	A_1_R
N6	EXT	−0.34	A_1_R
	HAB	−0.121	TNF-α
	EXT	−831^*^	TNF-α
**RECOVERY**			
Stage 3 sleep latency	HAB	0.295	A_1_R
RecN	EXT	−0.343	A_1_R
	HAB	−0.228	A_2A_R
	EXT	−0.251	A_2A_R
	HAB	−0.06	TNF-α
	EXT	−0.571^*^	TNF-α
Sleep onset latency	HAB	0.341	A_1_R
REcN	EXT	0.389	A_1_R
	HAB	−0.124	TNF-α
	EXT	−0.37	TNF-α

* *Significant correlations (p < 0.05)*.

*N6, the sixth night before the 24 h sleep deprivation*.

*RecN, the night of sleep recovery*.

## Discussion

Our present study shows that A_2A_R mRNA levels in leukocytes of healthy subjects increase after 24 h-*SD* and recover basal levels after a night of sleep recovery, while A_1_R has not changed, and this in two initial sleep conditions (21.00–07.00 and 22.30–07.00 bedtimes). The gene expression of P2RX_7_ is increased after the night of sleep recovery compared to 24 h-*SD* in both sleep conditions, while IL-1β and TNF-α were not changed after 24 h-*SD* and sleep recovery.

In this study, we analyzed gene expression in leukocytes because it may be a useful surrogate for gene expression in the CNS, when relevant genes are expressed in blood and brain (Chennaoui et al., 2005; Sullivan et al., 2006). The leukocyte gene expression of A_1_ and/or A_2A_ receptors, as that of P2X_7_ receptor and the pro-inflammatory cytokines TNF-α and IL-1β was demonstrated altered in various neurological pathologies or in the stress conditions of total sleep deprivation and sleep restriction; (Varani et al., 2010; Backlund et al., 2012; Chennaoui et al., 2014; Sauvet et al., 2015; Casati et al., 2016). For Hurtado-Alvarado et al. (2016), sleep loss may induce blood-brain barrier disruption through a systemic low-grade inflammation characterized by the release of several molecules including cytokines, adenosine, and hormones.

Sleep mechanisms are complex and multiple brain areas have been implicated in controlling the switch between wakefulness and the general state of sleep. Sleep-active neurons in the preoptic hypothalamus area inhibits wake-active neurons in the lateral hypothalamus (histamine neurons) and in the brainstem (serotonin and norepinephrine neurons). Sleep-active GABAergic neurons in the preoptic hypothalamus are inhibited by norepinephrine, acetylcholine and a subgroup by serotonin (Weber and Dan, 2016). In addition, adenosine, that accumulates during wakefulness under sleep pressure, has been extensively studied as a somnogen. Adenosine regulates also multiple physiological functions such as arousal, neuroprotection, learning and memory, cerebral blood circulation as well as pathological phenomena such as epilepsy. It is produced primarily from the metabolism of ATP and exerts pleiotropic functions throughout the body. Intra- and extra-cellular adenosine levels rise in response to physiological stimuli and with metabolic/energetic perturbations (such as prolonged wakefulness), inflammatory challenges, and tissue injury. In the CNS, several pieces of evidence support a role for the A_1_R and A_2A_R in mediating the sleep promoting actions of adenosine. In the human brain, a previous study evidenced that a single night of sleep deprivation leads to a significant increase of the A_1_R availability in cortical and subcortical brain regions (Elmenhorst et al., 2007), and there is any data on A_2A_R at the CNS or at the peripheral level during sleep deprivation. Our study is the first showing an increase in the gene expression of A_2A_R in leukocytes of healthy subjects after 24 h of sleep deprivation and a return to basal level after a night of recovery sleep. No change was observed in the gene expression of A_1_R. Another sleep regulatory signal is initiated by extracellular ATP changes that are detected by the purinergic P2X_7_ receptors on glia, and causes in turn the release of the somnogenic TNF-α and IL-1β pro-inflammatory cytokines (Krueger et al., 2008, 2010, 2013). Indeed, the ATP acting at P2X_7_ receptors was initially demonstrated to serve as an efficient secondary stimulus for the generation and release of IL-1β from proinflammatory cells (Ferrari et al., 1997). Krueger et al. demonstrated that mice lacking the P2X_7_ receptor exhibit reduced duration of non-REM sleep and reduced EEG δ wave power during non-REM sleep compared to control mice after sleep deprivation (Krueger et al., 2010). There is only one study showing increase of P2RX_7_ expression in monocytes of healthy subjects (eight participants, four males, and four females) after total sleep deprivation, and authors suggested that is a stress response to sleep deprivation (Backlund et al., 2012). In our study, we showed significant increase of P2RX_7_ mRNA after the night of sleep recovery compared to 24 h-*SD*, and no change IL-1β, and TNF-α neither after 24 h-*SD* nor after the night of sleep recovery. This does not preclude a TNF-α response to TSD, as we previously revealed increased whole-blood gene expression of TNF-α after 25 h of sleep deprivation and increased circulating levels at 34 and 37 h of sleep deprivation (Chennaoui et al., 2011, 2014). Also Irwin et al. have evidenced an increase of the spontaneous TNF-α monocytic expression after a night of sleep loss, which persisted in monocytes even after a night of recovery sleep (Irwin et al., 2006, 2015).

In the periphery, significant levels of the A_2A_ or P2X_7_ receptors expression have been evidenced in immune cells such as lymphocytes, monocytes, peripheral macrophages, neutrophils (North, 2002; Fortin et al., 2006; Fredholm et al., 2007), and it has become clear that they play critical role in the modulation of inflammatory reactions through complex mechanisms (Skaper et al., 2010; Sheth et al., 2014). Human neutrophils expressed significant amounts of A_2A_R transcripts that are maximally up-regulated after 4 h stimulation with low TNF-α concentration and maintained up to 21 h (Fortin et al., 2006). In this study, TNF-α also increased A_2A_R protein expression but the kinetic of induction is different from that of A_2A_R mRNA as the amounts of A_2A_R protein had returned to baseline after 12 h of TNF-α exposure. In line with most studies showing an increase in monocytic TNF-α expression during total sleep deprivation, increased expression of A_2A_R in our study (additionally in positive correlation with TNF-α) could be the result of low-grade inflammation and related TNF-α generated by the total sleep deprivation.

In our study, we determined if basal sleep duration influences A_1_R, A_2A_R, and P2RX_7_ mRNA levels and IL-1β and TNF-α during total sleep deprivation and sleep recovery, because down-regulation of A_1_R was suggested to mediate beneficial effect of sleep extension on cognitive performance during total sleep deprivation and recovery (Arnal et al., 2015). We observed higher total sleep time and higher duration time of N1, N2, and REM sleep stages in the extended basal sleep condition, but no effect on the expression of A_1_R which partially responds to our hypothesis (Arnal et al., 2015), no effect on A_2A_R, and P2RX_7_, nor on IL-1β and TNF-α. However, we did not measure their circulating protein level or function nor adenosine levels which limits the responses to our hypothesis (Arnal et al., 2015) and that of Rupp et al. who suggested that changes of extracellular adenosine/adenosine receptor ratio may explain behavioral consequences of sleep restriction, recovery, and sleep extension (Rupp et al., 2009). We confirmed that expression of A_1_R gene is low compared A_2A_R as previously highlighted in neutrophils (Fortin et al., 2006), and we can suggest thus that A_2A_R expression in leukocytes could be a reliable peripheral marker of sleep deprivation in human. Our results also show high and opposite correlations between A_1_R and A_2A_R expressions at baseline before sleep deprivation and latency of stage 3 sleep during the previous night, which give meaning to the suggestion of Sheth et al. (2014) concerning the involvement of adenosine receptors in the regulation of sleep. From their studies on mice with a global deletion in the p50 subunit of NF-κB, Sheth et al. (2014) speculated that the increase in cortical A_2A_R could explain the increases in slow wave and REM sleep, in addition to their rapid recovery following sleep deprivation. In our study, TNF-α expression was also negatively correlated with the latency of stage 3 sleep in the EXT sleep condition, which may emphasize Krueger et al. who suggested that TNF-α plays a role in sleep regulation and in particular enhances non-REM sleep (Krueger et al., 2008). Our results showed that high levels of stage 3 sleep latency coincided with high A_1_R (*r* = 0.703, *p* < 0.05) and low A_2A_R and TNF-α mRNA levels (*r* = −0.650, and *r* = −0.720, *p* < 0.05) in the EXT and HAB sleep conditions, respectively. In addition, these correlations were not significant when examined after the night of sleep recovery, which may be related to a buffering effect of the stage 3 rebound during the recovery night after TSD (Arnal et al., 2015). The correlations we observe could add information on the relationship between A_1_R and A_2A_R and TNF-α gene expression and stage 3 sleep which is the sleep stage that mediates at least some of the beneficial functions of sleep on the brain, such as memory consolidation (Tononi and Cirelli, 2014). We also consider that it would be interesting to study the effects of sleep deprivation and/or sleep extension on other neurotransmitters that play wake-promoting action such as norepinephrine and serotonin in the leukocytes from healthy subjects. Indeed, monoamine's receptors are expressed by leukocytes (Chennaoui et al., 2005), and sleep deprivation has been evidenced to increase serotonergic turnover in the basal forebrain (Zant et al., 2011) and to induce alterations in the norepinephrine system in the basal forebrain and cingulate cortex (Kim et al., 2013).

Some limitations of this study need to be noted. We focused on determination of peripheral gene expression of five biomarkers involved in the sleep regulation, but we did not include determination of their protein levels. Future studies are needed to determine whether sleep deprivation-induced changes in leukocyte gene expression are paralleled by altered protein levels. In addition, we conducted a highly controlled laboratory study with a small sample size of healthy young subjects, so future studies should be made on large healthy population or clinical cases with sleep disorders.

## Conclusion

The presented results showed for the first time that 24 h of sleep deprivation increased gene expression of the A_2A_R in leukocytes of healthy subjects and did not change A_1_R, and this even if bedtime was initially increased by 1.5 h per night for six nights. A delayed increase of the P2RX_7_ expression after the night of sleep recovery is observed, and TNF-α and IL-1β expressions were not changed. Interestingly the latency of stage 3 sleep was positively correlated with A_1_R and negatively with A_2A_R and TNF-α expression. The correlation analysis supported the hypothesis that adenosine receptors in the periphery may reflect sleep pattern in healthy subjects. Further, studies are warranted to determine if A_2A_ receptor changed at the protein level and functionality.

## Author's note

This work was performed in the French armed forces biomedical research institute (IRBA), Brétigny sur Orge, France.

## Author contributions

Authors have made substantial contributions to the following: Conception and design of the study: MC, PA, FS, and DG. Acquisition and analysis of data: MC, PA, CD, and FS. Interpretation of data and final approval of the version to be submitted: MC, PA, CD, DL, FS, and DG. Writing the manuscript: MC, PA, DL, FS, and DG. Revisiting the manuscript: MC, PA, DL, FS, and DG.

## Funding

At the IRBA (French Armed Forces Biomedical Research Institute, ministry of defense) studies are funded in operating cost by the DGA (General Directorate for Armament, ministry of defense), contracts 14Ca703 and PDH-1-SMO-2-508.

### Conflict of interest statement

The authors declare that the research was conducted in the absence of any commercial or financial relationships that could be construed as a potential conflict of interest. The reviewer RG and handling Editor declared their shared affiliation, and the handling Editor states that the process nevertheless met the standards of a fair and objective review.
